# Supplementary material for: Observer-based prescribed-time lag bipartite consensus of nonlinear multi-agent systems under event-triggered mechanism
Source: PLoS One. 2026 May 21;21(5):e0349879. doi: 10.1371/journal.pone.0349879 (PMC13193614; doi:10.1371/journal.pone.0349879)
Supplement: Attachment — Submitted filename: Response to Reviewers.docx [file pone.0349879.s007.docx]

**Authors’ Response to Editor’s and Reviewers’ Comments**

The authors wish to thank the Editor and the anonymous reviewers very much for their valuable comments and suggestions, which leads to significant improvement of the quality and presentation of the manuscript. In the following, we will explain how the editor’s and reviewer' comments have been taken into account in the revision.

**Response to Editor**

1. The revised manuscript has been formatted in strict accordance with PLOS ONE’s style and file naming guidelines.

2. All author-generated code to replicate the results of our study have been uploaded to the Supporting Information files as required.

3. The funders had role in study design, data collection and analysis, decision to publish, or preparation of the manuscript, and has attached the amended Role of Funder statement in cover letter.

4. All Funding Statements have been removed from the Acknowledgments section, and the amended statement has been included in cover letter.

5. All raw data required to replicate the results of our study have been uploaded as the Supporting Information files as required.

6. High-resolution and clearer versions of Figures 2, 3, and 4 have been newly uploaded.

7. The references recommended by the reviewers have been reviewed, and relevant ones have been cited in the manuscript.

**Response to Reviewer** **1**

**Comment 1:** The notation system and several basic definitions need substantial revision.

**Response 1:** **Thank you for your valuable advice and careful work!**

We have revised the notation system and basic definitions, unified all symbols, and optimized them to be accurate, complete and consistent with field conventions.

**Comment 2:** Theorem 2 invokes Assumptions 1, 2, and 3, but Assumption 3 is not explicitly stated in the provided preliminaries.

**Response 2:** **Thank you for your valuable advice and careful work!**

We apologize for the mistake in Theorem 2. Specifically, Theorem 2 only relies on Assumptions 1 and 2, and Assumption 3 is not involved in its proof. We have removed the incorrect citation of Assumption 3, and double-checked all theorem and lemma citations in the manuscript to ensure accuracy.

**Comment 3:** The prescribed-time design and the time parameters $T$, $T_{1}$, $T_{2}$, and $T_{3}$should be clarified much more carefully.

**Response 3: Thank you for your valuable advice and careful work!**

We clarify the time parameters related to our prescribed-time design as follows:

$T$ is the time constant specified by the user; $T_{1}$ and $T_{2}$ represent the prescribed convergence time of the position states and velocity states for the prescribed-time dynamic observer, respectively; $T_{3}$ denotes the prescribed convergence time of the closed-loop nonlinear system under the control protocol.

**Comment 4:** The main proofs, especially for Theorems 1 and 2, rely on several matrix inequalities and positive-definiteness claims, but the derivations are presented too briefly. In particular, the manuscript needs to justify more explicitly why the chosen Lyapunov functions are positive definite under the proposed parameter conditions, and how the bounds are transformed step by step.

**Response 4: Thank you for your valuable advice and careful work!**

In the manuscript, we have elaborated the proof procedures for Theorems 1 and 2. We have further added detailed explanations on the positive definiteness of Lyapunov functions and transformation of related matrix inequalities.

**Comment 5:** The Introduction would benefit from citing and discussing several references relevant to the research methodology, which could further enrich the background on nonlinear control and event-triggered control, such as 10.1016/j.automatica.2024.111511, 10.1016/j.automatica.2022.110792, and 10.1109/TIE.2026.3654623.

**Response 5: Thank you for your valuable advice and careful work!**

We have cited the three literatures in the Introduction, supplemented the relevant research background of nonlinear control and event-triggered control, and improved the completeness of the literature review accordingly.

**Response to Reviewer 2**

**Comment 1:** The contribution should be stated by comparing with the existing results, therefore, the novelty should be further improved and highlighted.

**Response 1:** **Thank you for your valuable advice** **and careful work!**

We have revised the manuscript accordingly. In the Introduction section, we have supplemented the innovations of this paper, analyzed the limitations of existing methods, and clarified the core advantages of the proposed method.

**Comment 2:** The reasonability and references of assumptions should be given.

**Response 2:** **Thank you for your valuable advice and careful work!**

In the manuscript, we have supplemented the rationality explanation and relevant literature support for all assumptions.

**Comment 3:** Some latest results about finite-time control should be introduced such as tuning function-based light computational adaptive fixed-time control for overhead cranes with multiple uncertainties, security-driven adaptive iterative learning formation control for multiagent systems etc., to make the review complete.

**Response 3:** **Thank you for your valuable advice and careful work!**

We have cited these two references in the Introduction section, supplemented the latest research results on finite-time control, and improved the completeness of the literature review.

**Comment 4:** Fig. 4 is unclear, please revise it.

**Response 4:** **Thank you for your valuable advice and careful work!**

In the manuscript, we have completely redrawn and optimized Fig. 4 to improve its clarity significantly. The revised figure is now clearer and more intuitive, which can better support the corresponding analysis and conclusions in the paper.

**Comment 5:** Some comparison results should be shown to explain the novelty of the proposed method.

**Response 5:** **Thank you for your valuable advice and careful work!**

Few existing works comprehensively take unknown leader information, communication congestion and nonlinearity into account. In addition, prescribed-time control combined with event-triggered mechanism under such conditions is rarely studied. Thus, direct comparison are unavailable. Our study extends the research scope, and additional theoretical analysis has been provided to verify its novelty.

**Comment 6:** The future work should be added to the conclusion.

**Response 6: Thank you for your valuable advice and careful work!**

We have added the future work in the conclusion section.

**Comment 7:** The linguistic quality or grammar problems need improvement, such as "$\beta$ are the observer gains" etc. The authors should pay attention to these problems.

**Response 7: Thank you for your valuable advice and careful work!**

We have carefully proofread and revised the entire manuscript, and further improved its linguistic quality and corrected grammatical problems.
